# Semiautomated 3D Root Segmentation and Evaluation Based on X-Ray CT Imagery

**DOI:** 10.34133/2021/8747930

**Published:** 2021-02-15

**Authors:** Stefan Gerth, Joelle Claußen, Anja Eggert, Norbert Wörlein, Michael Waininger, Thomas Wittenberg, Norman Uhlmann

**Affiliations:** ^1^Development Center X-Ray Technology (EZRT), Fraunhofer Institute for Integrated Systems (IIS), Flugplatzstraße 75, 90768 Fürth, Germany; ^2^Biomedical Engineering Department, Fraunhofer Institute for Integrated Systems (IIS), Am Wolfsmantel 33 11, 91058 Erlangen, Germany

## Abstract

**Background:**

Computed X-ray tomography (CTX) is a high-end nondestructive approach for the visual assessment of root architecture in soil. Nevertheless, in order to evaluate high-resolution CTX data of root architectures, manual segmentation of the depicted root systems from large-scale volume data is currently necessary, which is both time consuming and error prone. The duration of such a segmentation is of importance, especially for time-resolved growth analysis, where several instances of a plant need to be segmented and evaluated. Specifically, in our application, the contrast between soil and root data varies due to different growth stages and watering situations at the time of scanning. Additionally, the root system itself is expanding in length and in the diameter of individual roots.

**Objective:**

For semiautomated and robust root system segmentation from CTX data, we propose the *RootForce* approach, which is an extension of Frangi's “multi-scale vesselness” method and integrates a 3D local variance. It allows a precise delineation of roots with diameters down to several *μ*m in pots with varying diameters. Additionally, *RootForce* is not limited to the segmentation of small below-ground organs, but is also able to handle storage roots with a diameter larger than 40 voxels.

**Results:**

Using CTX volume data of full-grown bean plants as well as time-resolved (3D + time) growth studies of cassava plants, *RootForce* produces similar (and much faster) results compared to manual segmentation of the regarded root architectures. Furthermore, *RootForce* enables the user to obtain traits not possible to be calculated before, such as total root volume (*V*_root_), total root length (*L*_root_), root volume over depth, root growth angles (*θ*_min_, *θ*_mean_, and *θ*_max_), root surrounding soil density *D*_soil_, or form fraction *F*. *Discussion*. The proposed *RootForce* tool can provide a higher efficiency for the semiautomatic high-throughput assessment of the root architectures of different types of plants from large-scale CTX. Furthermore, for all datasets within a growth experiment, only a single set of parameters is needed. Thus, the proposed tool can be used for a wide range of growth experiments in the field of plant phenotyping.

## 1. Background

A nondestructive investigation of root systems in soil grown under controlled conditions or on the field is essential to understand plant growth in order to increase the crop yield or to adapt to changing climate conditions [1, 2]. Nondestructive 3D volume imaging approaches, e.g., those based on computed X-ray tomography (CTX), have proven to be quite suitable methods to capture, visualize, and analyze root system architecture [1–7]. Using controlled growth conditions within pots, a submillimeter resolution of the roots can be achieved using such volumetric scanning devices [3]. Nevertheless, with increasing spatial resolution of CTX data and hence related better image quality, the amount of image data to be analyzed also increases. Thus, with the rising demand for nondestructive 3D imaging of the below-ground area, the amount and size of the related datasets become challenging and increase the need for advances in automated root phenotyping [1, 5, 7, 8].

In previous work [4], we presented a comparison of manual approaches for root segmentation and measurement based on CTX image data. The scanning approach that we applied yielded high-quality data with respect to successive manual segmentation and showed a clear root architecture for all tested pot sizes. The manually segmented root structures were compared to total root length computed with WinRHIZO [9, 10] after the harvest. Root traits calculated from CTX images showed a reduced total root length compared to that found with WinRHIZO. One of the well-known causes for these effects of bad visibility of roots (and consequently bad segmentation) is that X-ray imaging is based on photon transmission through the samples which is mainly dependent on the density of the observed materials. Therefore, the resulting attenuation coefficients for roots and the surrounding soil can be very similar and can be difficult to distinguish visually [2]. This difficulty increases for fine lateral roots due to their small diameter.

In order to improve such segmentation approaches based on large-scale CTX data with respect to available ground truth and to support the users with automated image analysis, an extension of the “multi-scale vesselness” approach has been implemented. This approach for the automated description of human vessel-trees was suggested by Frangi et al. [11]. The CTX data has been obtained from bean and cassava plants.

## 2. Objective

As manual root segmentation from volumetric imagery such as CTX is time consuming, error prone, and not always reproducible, automatic or semiautomatic segmentation approaches are necessary and helpful. To this end, various tools have been proposed in the past in literature.

### 2.1. Related Work

Most work known in the field of plant-root segmentation is based on the aforementioned original work of Frangi et al. in 1998 [11] who made use of the Eigenvalues from the Hessian matrix in order to describe the second order local structures of 2D or 3D images (also denoted as “vesselness”). Extensions to the work of Frangi et al. are, e.g., the works of Lo et al. in 2010 [12] or Schulz et al. in 2012 [13]. For example, Lo et al. [12] used the proposed multiscale cost-function suggested by Frangi et al. to systematically extend the vessel-trees by detecting branches with locally optimal paths by including geometric characteristics of the vessel-tree. Similarly, Schulz et al. [13] detected tubular structures and connected them using the *A*^∗^ algorithm for the shortest path estimation. Hence, paths within root candidates are preferred over paths in soil [13]. For the segmentation of human vessel-trees in data volumes, e.g., CTX, Jerman et al. in 2015 [14] proposed the weighted use of the extracted Eigenvalues of the Hessian matrix originally suggested by Frangi et al. Using an alternative approach from computer vision, e.g., Mairhofer et al. in 2016 [15] proposed a “virtual tracking” approach based on the well-known level set segmentation method, which is guided slice-by-slice in a tracking manner by grayscale intensities and shape information on the boundaries. Also, Gao et al. [16] made use of local features detected in the tubular shapes of roots to reconstruct the root architectures from CTX data.

Recent developments from the field of machine learning using deep convolutional neural networks (DCNNs) allow the use of so-called “U-Nets” for the 3D-segmentation of root architectures as recently suggested by, e.g., Smith et al. [17] or Zhao et al. [18]. In a similar way, Soltaninejad et al. [19] also proposed an encoder-decoder architecture for 3D root segmentation. Nevertheless, in contrast to all other methods known for root segmentation which rely on the algorithmic application of knowledge about root geometry and growth, e.g., [11–16], for deep learning approaches [17–19], a lot of manually presegmented image data is necessary in order to train the used neural networks.

### 2.2. Contributions

Based on these observations, we developed a semiautomatic parameter-based segmentation approach (termed “*RootForce*”) for the delineation of root architectures depicted in CTX data, which can easily be adopted and parametrized to various root types, as described in Section 3.3. *RootForce* is especially designed for high-throughput time series of CTX data (3D + *t*) to be used with one single parameter set *P* for the complete time series. By using one single parameter set *P* for all acquired data of one plant over time, all achieved segmentations can be compared to each other; thus, the root growth of such a plant can be objectively measured over time. Such high-throughput CTX data (with a scan volume of 20 × 20 × 20 cm and up to a cubic voxel size of 75 *μ*m) can, e.g., be acquired within seven minutes per pot. Nevertheless, with these scanning possibilities, the proposed algorithm has to deal with low signal-to-noise ratios and must yield stable segmentation results with varying soil conditions (soil moisture, soil compactness) over time.

We compare the automated segmentation of root architectures depicted in CTX data using *RootForce* to manual root segmentation. Additionally, we made a segmentation comparison with the *Rootine* tool published by Gao et al. [16] on both plant types. The ground truth achieved with WinRHIZO [9, 10] was made for the bean plant experiment. As *RootForce* was designed and implemented to be adaptable to various root types, we will address root segmentation for two different root types and research aspects:

- For bean plants, we evaluate high-resolution scans (with a cubic voxel size of down to 28.15 *μ*m) for detailed root analysis in comparison to manual segmentation and WinRHIZO [9, 10] data, and relate the results of delineated root length and compare them with manual segmentations from previous results [4] (cf.  Section 4.1)
- For cassava plants, we describe and analyze a time series over 100 days with consecutively obtained CTX scans. We observe the extracted root traits over time and also compare them to manual segmentation of the root architectures with a focus on storage roots and growth rate (cf.  Section 4.2)

## 3. Methods

### 3.1. Plant Material

Two types of plant-root data were used for the experiments, namely, bean and cassava plants.

Common bean plants (*Phaseolus vulgaris* L. aka. “Shiny Fardenlosa”) were grown from seeds in a growth chamber in a mixture of homogenized agricultural topsoil and coarse sand [4]. The unspecified soil (73% sand (mostly fine), 23% silt (mostly coarse), and 4% clay (extracted from a field close to Forschungszentrum Jülich) [20]) was filled into PVC tubes of two different sizes, namely, with inner diameters of (a) ∅ = 34 mm (small pot: a height of 200 mm and a volume of 0.182 l) and (b) ∅ = 56 mm (medium pot: height 200 mm, volume of 0.49 l). The pots were watered, and after water drainage, the seeds were laid down in 20 mm deep holes and covered with soil for germination; the pots were watered automatically once per day with tap water. The growth chamber was set to 16 h light/8 h dark and 20°C/16°C, respectively, while relative humidity was kept constant at 60 ± 3%.

For the growth (3D + *t*) experiments over time, three different cassava genotypes (TME419, TMS 30572, and 980581) with five plants each were grown from tissue culture and then transferred into pots with an inner diameter of ∅ = 200 mm and 150 mm height with sieved (5 × 5 mm^2^ grid) potting mix (Einheitserde classic ED73 https://www.einheitserde.de). The plants were stored over 100 days in Conviron A1000PG growth cabinets with 50% humidity and temperatures of 28°C and 25°C during daytime (12 h) and nighttime (12 h), respectively. However, one of the genotypes was additionally used within another measurement campaign, and so the robustness of one parameter set *P* could be demonstrated on up to 500 different plants.

### 3.2. Scanning

For image acquisition, two CTX systems at the Fraunhofer Development Center X-Ray Technology (EZRT) [21] were utilized.

#### 3.2.1. Bean Plants

Bean plants were scanned 23 days and 24 days after seeding. For the measurements of the small pot, the cubic voxel size was 28.15 *μ*m; for the medium pot, the voxel size was 34 *μ*m. The CTX setup consists of an FXE 225.99 X-ray tube and a PerkinElmer XRD 1620 flat panel detector and operates with a frame rate of 2 images per second in 14-bit full-frame mode and yields projection images of 2048 × 2048 pixels. Since some planting pots were up to 300 mm in height, multiple scans of the samples were made at different heights. The obtained individual volumes were merged afterwards to obtain a complete recording of the plants. More details of the scanning process can be found in [4]. The tube parameters were set to 220 kV voltage, 180 *μ*A current, and 499 ms exposure time to ensure an optimal image quality. Scans were made with continuous sample rotation (fly-by) over 360° and 1200 projections resulting in a total scan time of approximately 10 minutes per plant per height.

#### 3.2.2. Cassava Plants

The cassava plants were regularly scanned on Monday, Wednesday, and Friday morning over 100 days. The second CTX setup consists of a Comet MXR-225HP/11 X-ray tube and a Fraunhofer XEye 2020 flat panel detector and operates with a frame rate of 2.85 images per second in 16-bit full-frame mode and yields projection images of 2040 × 2048 pixels. The settings used for the scans and the reconstruction are as follows. The acceleration voltage of the tube was set to 175 kV and the current to 4 mA with a Cu prefilter of 1 mm allowed to irradiate a pot with a diameter of 200 mm with a sufficient ratio between transmission and absorption (about 10% transmission behind the pot). On the X-ray detector, an exposure time of 350 ms was used for each of the 1600 projections during the 360° rotation resulting in a total scan time of 9 minutes for each plant. Thus, the effective dose to the plants during a single scan is around 200 mGy. The scans were conducted with an optical magnification of 1.14 resulting in a cubic voxel size of 87.7 *μ*m. Nevertheless, to reduce the data size and increase the speed of downstream analysis, the data was reconstructed on a cubic voxel size of 175 *μ*m.

Based on the spatial voxel sampling of the used scanning systems, the detectable minimum root diameter is naturally limited. For the low-contrast scenario of roots in soil, the typical minimum detectable root diameter is about 2.5 voxels (approx. 0.4 mm) in diameter. All parameter settings of the image acquisition are listed in Table 1 below.

### 3.3. Semiautomatic Segmentation with *RootForce*

Based on the findings from previous work and preliminary studies [4, 11–16], we developed a semiautomatic tool named *RootForce*. As mentioned above, the proposed segmentation approach is partially based on the idea of “multi-scale vesselness” as suggested by Frangi et al. in 1998. This approach has been originally designed to detect tubular structures in volumetric medical CT image data using a Hessian Eigenvalue analysis.

**Figure 1:**
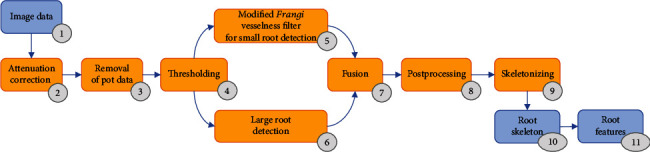
Workflow of the proposed semiautomatic segmentation.

Our approach is in some parts similar to the work of Gao et al. [16], but with some necessary modifications due to the thickness of the storage roots and with respect to the low signal-to-noise ratio observed in our data. This lower SNR is related to the increased scanning speed for the acquisition of high-throughput volume data in our work.  Figure 1 provides the core workflow of our approach. The proposed workflow starts with the image data (Figure 1 (1)). Similar to other works [12, 13, 16], the algorithm detects roots and side-roots based on their different attenuation coefficients compared to the surrounding volume (Figure 1 (2)). This step also includes the normalization between the attenuation coefficient [22] and the gray values. In order to reduce the volume of the data for further processing, as well as to eliminate unnecessary information such as the data of the air space, the pot and some surrounding soil are removed (Figure 1 (3)). The removal of the pot data is only dependent on the pot thickness and the attenuation coefficient of the pot. A final preprocessing step (Figure 1 (4)) includes automated thresholding of data containing roots in the remaining soil, in order to adequately prepare the volume data for Frangi's modified vesselness approach and the homogeneity calculation. The thresholding consists of an upper and a lower limit of attenuation coefficients (*A*_min_, *A*_max_). The attenuation range (*A*_min_, *A*_max_) depends on the type of soil used within the experiment as well as the type of plants under consideration. At this point, similar to [16], two different calculations are done in parallel. Step 5 is Frangi's modified vesselness approach (Figure 1 (5)), and step 6 is related to the variance calculation for large roots (Figure 1 (6)). This modified filter considers the shape and geometry of the root data. It segments a connected root system from the thus far preprocessed CTX volume.

The multiscale calculation or the vesselness measure (Figure (5)) is computed as a function of the Hessian matrix. Particularly, the Eigenvalues (*λ*_1_, ⋯, *λ*_3_) with *λ*_1_ > *λ*_2_ > *λ*_3_ describe the local neighborhood of a voxel. If bright vessels on a dark background are to be detected, these Eigenvalues must have the relationships *λ*_1_ ≈ 0 and 0 ≫ *λ*_2_ ≈ *λ*_3_; see Frangi et al. [11]. This “vesselness” is computed in 3D on the whole CTX volume neglecting absorption values above and below a distinct attenuation coefficient range (*A*_min_, *A*_max_). Additionally, depending on the plant type, the sensitivity for different root diameters *D*_root_ can be specified within our approach.

However, when searching for larger root structures, namely, tubers or storage roots, the original vesselness defined by Frangi et al. [11] is not very significant anymore. In contrast to Gao et al. [16], we extended *RootForce* for larger root structures based on their 3D homogeneity (Figure 1 (6)). Specifically, larger roots are detected using the computation of a local 3D variance calculation which is computed utilizing a fast 3D-Gaussian filter implementation, were the local variance is defined as follows:

(1) $$\begin{array}{c} V_{\text{local}}\left(x\right)=\frac{1}{\sqrt{2\pi \lambda^{2}}}\;\exp\left(-\frac{{\left(x^{2}-{x_{0}}^{2}\right)}^{2}}{2\lambda^{2}}\right)-{\left(\frac{1}{\sqrt{2\pi \lambda^{2}}}\exp\left(-\frac{{\left(x-x_{0}\right)}^{2}}{2\lambda^{2}}\right)\right)}^{2}, \end{array}$$

where *x*_0_ and *x* denote voxels in a 3D space.

The consecutive step (Figure 1 (7)) merges the small and large detected vessel structures from both branches (Figure 1 (5) and (6)) for a joint segmentation result. The merging process is based on two different thresholds (*θ*_1_ and *θ*_2_) selecting the limits for small and large structures appearing in the merged segmentation. For postprocessing (Figure 1 (8)), an optional volumetric nonlinear median filter can be used on the fused 3D data, whose kernel size can be selected. Furthermore, an optional labeling step can be applied combined with a size filter in order to eliminate remaining small superfluously and unconnected root fragments.

By the key parameters, *P* being the attenuation coefficient range (*A*_min_, *A*_max_), the root diameter *D*_root_, and the merging parameters (*θ*_1_ and *θ*_2_) for small and large structures, the proposed semiautomatic approach can be adapted in order to differentiate plants and various root types. Hence, it is suited for the segmentation of plants with very fine roots such as wheat or maize as well as for plants with storage roots like cassava or potato.

After a final skeletonizing step (Figure 1 (9)), the algorithm determines distinct root traits utilizing a so-called “*Reeb Graph*” approach [23, 24] (Figure 1 (10)).

Furthermore, characteristic features (traits) such as biomass *B*_root_ over depth as profile or global features such as root volume *V*_root_; root length *L*_root_; minimum, maximum, and mean root angles *θ*_min_, *θ*_max_, and *θ*_mean_; biomass quantiles over depth; form fraction *F*; mean root density; mean soil density; relation between soil and root density; density of soil around roots; number of root tips; and the root structure as *RSML* (root system markup language [25]) can be calculated (see Table 2 and Figure 1 (11)) using *RootForce*.

**Figure 2:**
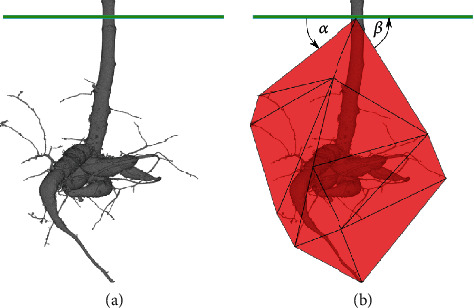
(a) Segmentation of a cassava plant. The green line represents the 3D soil-air interface, which indicates the entry point of the plant in the soil. (b) In red, the 3D convex hull of the segmentation is depicted with *θ*_min_ = *α* as the minimum and *θ*_max_ = *β* as the maximum root angle between the convex hull faces and the green soil slice.

For the computation of the total root volume *V*_root_, all root voxels are summed up and multiplied with the cubic voxel volume. For the root length *L*_root_, all voxels from the calculated *Reeb Graph* are summed up and the voxel edge size is used to obtain the full root length. Based on the calculated total root volume *V*_root_, it is possible to calculate the root volumes for each slice in the reconstruction (height-direction) which leads to the quantiles for the root volume over depth for the plant. As form fraction *F*, the total root volume *V*_root_ is divided by the volume of the root system 3D convex hull [26]. Based on the calculated convex hull, we use the top faces of the hull to define the minimum *θ*_min_ and maximum *θ*_max_ root angles between the faces and the soil, see Figure 2.

The density *D* is approximated for each voxel as product of the gray value and the correlated attenuation coefficient. Mean densities for roots and soil are the summed-up densities for each of the related voxels and divided through the number of the root or soil voxels. The relation of these two densities provides the fraction of both. For calculating the density of soil around soil, we sum up all densities of the voxel which are in a given radius around the segmented roots and calculate their mean value. Also based on the *Reeb Graph*, the value of root tips *T*_root_ is related to the number of all nodes of the graph not having any output edges.

The segmented root system can finally be saved as an RSML file (root system markup language [25]), which can be read with every software able to read and load RSML files.

Depending on the working computer and CTX data size, *RootForce* needs between 5 and 30 minutes to generate the root structure (applying a computer with 64 GB memory and using volume image data sizes up to 16 GB). These computation durations are many times faster than a pure manual segmentation and hence make preoperative binning redundant for the image segmentation. Thus, the computation can be performed on full spatial resolution. Furthermore, *RootForce* can operate autonomously in a batch mode, therefore allowing the evaluation of a complete set of measurement series at once.

The parameters used in the experiments can be found in Table 3.

#### 3.3.1. Bean Roots

The bean roots were processed automatically using the *RootForce* approach with a processing time of 15 minutes and 26 minutes on a system with 64 GB RAM and 32 GB RAM, respectively. The parameter for *RootForce* is the attenuation value range = (0.11, ⋯, 0.24) with root diameters *D*_root_ set to 2, 2.5, 3, 3.5, 4, 5, and 6 voxels. Out of the attenuation value area, an upper threshold and a lower threshold are calculated and used for the vesselness calculation. These values are manually adapted once to the pot, the soil, and the plant used within an experiment. For the small pot size, the lower first threshold was set to *θ*_1_ = 400 and the second threshold was set to *θ*_2_ = 2500. For the medium pot size, the first threshold was set to *θ*_1_ = 600 and the second threshold was set to *θ*_2_ = 2000. As minimum root volume, *V*_min_ = 1 mm^3^ for the small pot with 28.15 *μ*m resolution, and *V*_min_ = 1.6 mm^3^ was used for the medium pot with 34 *μ*m resolution.

#### 3.3.2. Cassava Datasets

The cassava datasets (#2 to #4) were processed automatically using the *RootForce* approach in binning mode (with a maximum processing time of 20 minutes on a system with a 32 GB RAM, a Quad-Core CPU 3.8 GHz, and no GPU support), resulting in a resolution of 175 *μ*m cubic voxel size. The resolution is comparable to other investigations on this field; for example, Colombi et al. in 2017 [3] stated a visual detection limit for roots at a diameter of around 175 *μ*m and 300 *μ*m. The parameter settings of *RootForce* were adapted for sieved soil resulting in an attenuation coefficient range = (0.08, ⋯, 0.2) and root diameters of *D*_root_ = 1, 1.5, 2, and 3 voxels to extract the root structure. *θ*_1_ = 60 was used for the first threshold and *θ*_2_ = 65,000 was used for the second threshold to completely fill the storage roots. The minimum root volume was set to *V*_min_ = 5 mm^3^.

### 3.4. Manual Segmentation

For manual root segmentation, the Volume Player Plus (VPP) software (Fraunhofer EZRT, Fürth, Germany) and the Modular Algorithms for Volume Images (MAVI) software package (Fraunhofer ITWM Kaiserslautern, Germany) were applied [27, 28]. In the first step, the datasets were binned using subvolumes 2 × 2 × 2 to a new resolution with a cubic voxel size of 56.3 *μ*m for a pot size with an inner diameter of ∅ = 34 mm, and a cubic voxel size of 68 *μ*m for a pot size with an inner diameter of ∅ = 56 mm. Afterwards, the pot material was removed from the data by manually fitting an ellipse to the inner pot wall. For root interactive segmentation, the so-called morphological hysteresis binarization in the range of the root gray intensity values was used. Therefore, for every dataset, the gray value area which characterizes the roots was determined. The range of possible root values is quite wide due to the different thickness of the roots. Large roots show higher gray intensity values than small ones. Based on empirical knowledge, the range of gray intensity values was limited by defining different gray intensity value ranges for small, middle, and big roots. In summary, the whole spectrum of gray intensity values depicting roots was covered but split into three parts linked to root thickness. Gap closures are used to generate a connected root architecture.

In the following steps, the datasets were cleaned from the remaining nonroot parts using the MAVI software. Stones, soil, and air- or water-filled pores are removed based on size and shape, and if they are not connected to the root system. For defining the connectivity of the objects, a 3D-neighborhood system is used. More details of the removal of nonroot parts can be found in [28]. The whole procedure for manually segmenting the root systems takes approximately two hours for each dataset.

### 3.5. Root Length Measurement

For comparison, the roots of the bean plants were finally excavated, washed, and scanned using WinRHIZO [9, 10] as a widely used standard technique. Parts of the WinRHIZO data was collected in the previous experiments and is described in [4].

### 3.6. Comparison to Other Segmentation Algorithms

For comparison with other common segmentation tools for CTX data, one bean plant and one cassava plant were also analyzed with *Rootine*, a software published by Gao et al. [16].

## 4. Results

### 4.1. Bean Plants

The roots of the bean plants were segmented manually as well as semiautomatically with the described *RootForce* approach in full spatial resolution as well as with the downsampled mode with 2 × 2 × 2 binning. Downsampling yielded a cubic voxel size of 56 *μ*m for a pot size with an inner diameter of ∅ = 34 mm (small pot), 68 *μ*m for a pot size with an inner diameter of ∅ = 56 mm (medium pot). Additionally, for the small and medium pot sizes, the depicted root system is also compared to the WinRHIZO data from previous experiments [4].

#### 4.1.1. Small Pot Size

**Figure 3:**
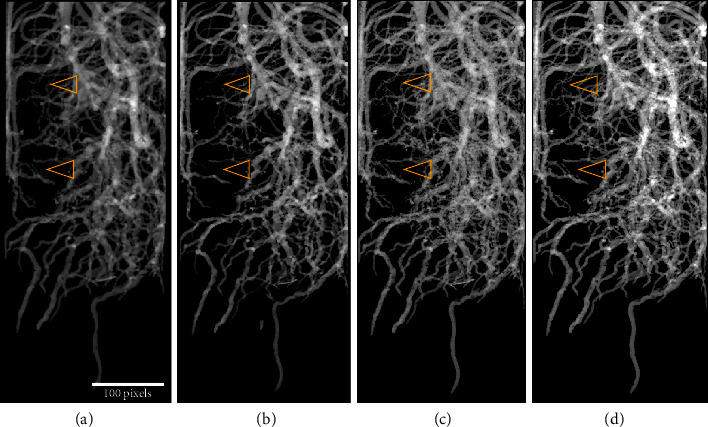
Projection images of a bean root system in a small pot with an inner diameter of ∅ = 34 mm and a height of 200 mm 23 days after sowing. (a) Manually segmented root system with downsampled images. (b) Same root system segmented with *RootForce* on downsampled images as in (a). (c) Root system segmented automatically by *RootForce* on full resolution. (d) Root system segmented automatically by *RootForce* and postprocessed with a nonlinear filter and fragment removal on full resolution. Arrowheads denote the same lateral roots in all images.

Figure 3 depicts maximum intensity projection (MIP) images of the roots segmented for the bean plant in the small pot. Figures 3(a) and 3(b) depict the manual and semiautomatic segmentation (obtained with *RootForce*) on downsampled images. In comparison, the main root system in both segmentation approaches is almost identical in both images. Only minor variations can be seen for the segmented lateral roots, as indicated by arrowheads for two examples. Nevertheless, the manual segmentation additionally visually depicts some root fragments not connected to the main root system, whereas the semiautomatic segmentation offers “better connection” (meaning less fragments and more connected parts) between the lateral roots and the main root system.

In contrast to manual segmentation, the described semiautomatic *RootForce* segmentation (see Section 3.3) can easily be conducted in full spatial resolution with a cubic voxel size of 28 *μ*m. Thus, the *RootForce* segmentation based on the original high-resolution data renders visible the lateral roots in the small pot data, as depicted in Figure 3(c). These lateral roots can be delineated in the semiautomatic processing, but it becomes more difficult to differentiate between lateral roots and soil (see arrowheads in Figure 3(c)).

Hence, a 3 × 3 × 3 volumetric median filter as a postprocessing step after the segmentation can help to keep lateral roots connected to the main root system and separated from the background soil. Also, in the postprocessing step, the remaining single soil fragments are removed using a labeling process combined with an object-size filter. A resulting postprocessed maximum intensity projection image is shown in Figure 3(d).

In summary, for roots grown in the small pots, the segmentation results of manual and semiautomatic processing are comparable, whereas semiautomatic processing is more precise as the high-resolution data allows the delineation of lateral roots down to 0.03 mm in diameter.

Another noticeable factor is that semiautomatic segmentation needs much less processing time (15 minutes with 64 GB RAM, 26 minutes with 32 GB RAM) than manual assessment (approximately two hours if the segmentation parameters are known) and is furthermore free from individual influences and thus reproducible.

#### 4.1.2. Medium Pot Size

**Figure 4:**
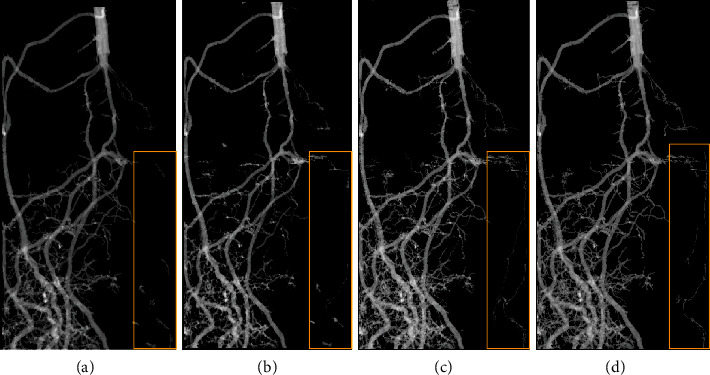
Maximum intensity projection images of a bean root system in a medium pot with an inner diameter of ∅ = 56 mm and a height of 200 mm 24 days after sowing. (a) Manually segmented root system with a reduced cubic voxel size of 68 *μ*m. (b) Same root system as (a) automatically segmented, also in reduced mode. (c) Automatically segmented root system using *RootForce* on full-resolution volume data with a cubic voxel size of 34 *μ*m. (d) Automatically segmented root system using *RootForce* and postprocessed root volume from (c) to remove background soil. Boxes denote the same side roots in all images.

Figure 4 shows maximum intensity projection images of the roots segmented for the bean plant (in the medium-sized pot with an inner diameter of ∅ = 56 mm). The comparison of manual and semiautomatic segmentation in Figures 4(a) and 4(b) after downsampling with 68 *μ*m spatial resolution also shows more segmented lateral roots as well as a better connection among them using semiautomatic root segmentation. Thus, semiautomatic delineation can be considered more reliable in contrast to manual segmentation.

In the segmentation based on the full data resolution depicted in Figure 4(c), especially the thin lateral roots are detected more clearly (indicated by the boxes drawn in Figure 4). Nevertheless, the amount of the remaining soil is also increased. Applying a nonlinear (3 × 3 × 3) median filter to separate the remaining soil from the root system also leads to the loss of some lateral roots, compare Figure 4(d). Thus, it can be stated that the recognition of lateral roots becomes more difficult at lower resolution.

#### 4.1.3. Comparison of *RootForce* with WinRHIZO and *Rootine*

The acquired root traits (Table 4) are compared with the root traits calculated from CT images of the segmented volumes shown in Figures 3(a) and 3(b) and Figures 4(a) and 4(b) for the small pots; 114% of the related root length *L*_root_ (obtained from WinRHIZO) has been computed from the semiautomatic segmentation, whereas manual segmentation results only in 69% of the root length. The higher value from semiautomatic segmentation is due to the higher amount of detected lateral roots and the better connectivity between them, as attached roots will be lost for root length calculation if the resulting gap to the main root system is too large. The segmented root system for a small pot size has no clear starting point in the recording, so a slight oversegmentation of the root length is caused by some ghosting artifacts in the upper part of the pot.

In contrast to the root length, the total root volume *V*_root_ calculated from manual segmentation is higher than that for the semiautomatic delineation. The reason for this can be found in manual processing: in order to keep the root system connected, the remaining soil is separated from roots by a morphological opening function followed by morphological closure. These opening and closure operations modify the root thickness and thereby the related root volume, whereas the semiautomatic segmented root volume remains unfiltered.

The calculated root traits for the medium-sized pot, also shown in Table 4, yield 83% of the root length obtained by WinRHIZO for the semiautomatic segmentation, in comparison to 57% for the manual one. As the lower slices of the medium pot (approx. 30 mm) have been missing in the data due to water standing in the bottom of the pot, 100% of WinRHIZO data is not achievable.

Again, for manual segmentation, morphological opening and closing operators were used to separate roots from the soil. These postprocessing steps lead to a distortion of root thickness, hence resulting in a comparable total root volume for manual and semiautomatic segmentation in the medium-sized pot at less root length.

**Figure 5:**
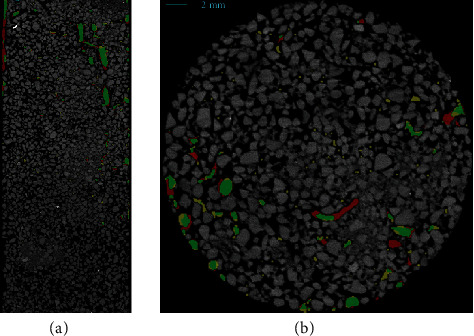
Comparison of the proposed *RootForce* segmentation with the reference *Rootine* segmentation [16]. Two slices from a bean plant in a small pot. Roots segmented by both approaches are marked in green, roots segmented only by *Rootine* are marked in yellow, and roots segmented only with *RootForce* only are marked in red.

For the comparison with *Rootine* published by Gao et al. [16], we used the bean plant in the small pot. Differences between the resulting segmentations of this comparison are depicted in Figure 5. Roots marked in green are segmented with both approaches, roots marked in yellow have only been segmented with *Rootine*, and roots marked in red have only been segmented with *RootForce*. Mainly, these segmentations look similar, but there are some roots which were not segmented with *Rootine* but with *RootForce*. The other way around, all roots segmented with *Rootine* were also segmented with *RootForce*. The other difference is that small pieces of soil are still segmented with *Rootine.* Via adjusting the thresholds, it was not possible to separate these remaining soil particles while keeping the root system.

#### 4.1.4. Additional Calculated Traits Using *RootForce*

The automated analysis with *RootForce* offers some further details to characterize the segmented root volumes in an automated manner, shown as global features in Table 2. These global features include main traits like root volume *V*_root_, root length *L*_root_, and form fraction *F*, supplemented by root biomass distribution over depth and detailed information about root angle, density, and relation of earth to root.

**Figure 6:**
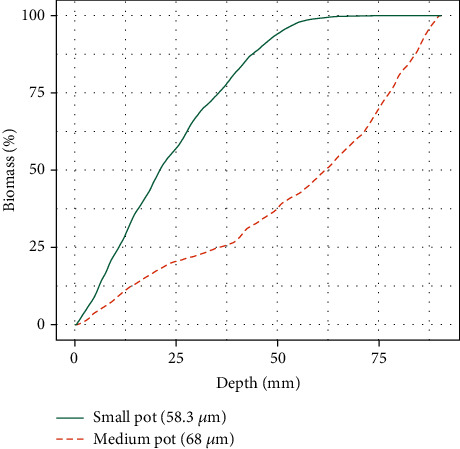
Root biomass *B*_root_ (in %) over depth (in mm) as a cumulative distribution for small- (green solid line) and medium- (orange dotted line) sized pots over the depth of the pot. Both datasets were extracted with *RootForce* in binning mode.

The root biomass *B*_root_ is computed with respect to the depth in the soil to show the distribution of root mass inside a pot. The value of *B*_root_ is depicted in Figure 6 as percentage of the cumulative distribution for the small- (green solid line) and medium- (orange dashed line) sized pots. These results are related directly to the maximum intensity projection images in Figures 3(b) and 4(b), depicting the semiautomatic segmentation of the roots with *RootForce* in binning mode. In the small pot, the roots are mainly depicted in the upper part and run out deeper into the pot at approximately 60 mm depth. In contrast, the medium pot shows a clear main stem with only a few lateral roots in the upper part. Most of the lateral and side roots are located at the lower part of the pot and grow to its bottom. This is also represented in the diagram by the orange dashed curve. Hence, this effect can be also be seen in Table 2 via the Rootmass quantiles. The 50% root biomass quantile is at 20.3 mm depth for the small-sized pot and at 62 mm depth for the medium-sized pot. The form fraction *F* is calculated as the total root volume *V*_root_ below the soil-air interface divided by the volume within a 3D convex hull of the root system. Therefore, this is a measure of the compactness of the root system architecture.

### 4.2. Cassava Plants

**Figure 7:**
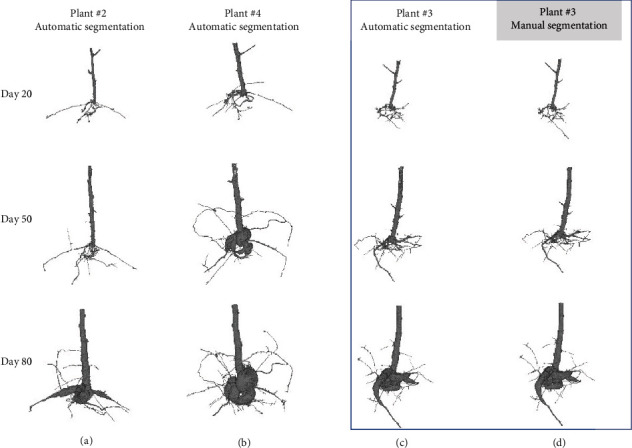
Semiautomatically (a–c) and manually (d) segmented 3D volumes of cassava root systems from genotype TME419 grown in pots with sieved soil with an inner diameter of ∅ = 200 mm and a height of 150 mm. Segmentation was obtained on a cubic voxel size of 175 *μ*m. For comparison, plant #03 was additionally segmented manually (d). The 3D images show the extracted root volume 20, 50, and 80 days after planting.

Besides the volumetric analysis of fully grown bean plants, the root development over time is examined for cassava plants in order to investigate in situ root growth parameters. For the investigated set of plants, the proposed semiautomated approach was able to segment a total of 708 measurements of cassava plants with the same parameter set for the algorithm, even for different moisture contents and time points. Furthermore, all three genotypes and all 5 replicates of each genotype measured during the whole experiment shared a common and constant set of parameters *P*. As an example, Figure 7 shows the segmentation results as 3D images for the three plants (#2, #3, and #4) of one genotype at days 20, 50, and 80 after planting. As the main stem of TME419 plant number #01 kinked and the storage root bulking was two months delayed compared to the other plants, this plant was not taken into account.

All 708 measurements have been analyzed with a constant set of parameters *P* once adjusted on the first scan of two plants. For comparison, plant #03 was also additionally manually segmented and is listed in Figure 7(d). All three plants show a clearly visible storage root development where plants #02 and #03 appear quite similar. The storage roots for plant #04 are significantly thicker.

#### 4.2.1. Comparison of *RootForce* and *Rootine*

**Figure 8:**
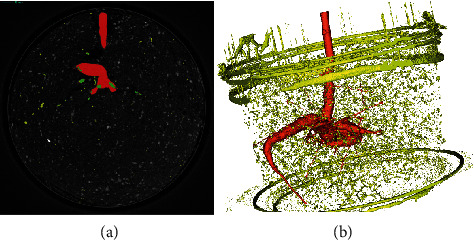
Comparison of *RootForce* and *Rootine* segmentation results. Roots marked in green are segmented with both algorithms, roots marked in yellow are only segmented with *Rootine*, and roots marked in red have only been segmented with *RootForce.* (a) Cross section of the measured dataset with a colored overlay. (b) 3D-vizualization of both segmentation as 3D mesh.

The segmentation results of CTX data using the proposed *RootForce* approach and the reference *Rootine* software are for storage roots of cassava plants that are quite different. Some cross sections are shown in Figure 8. Roots marked in green are segmented with both algorithms, roots marked in yellow are only segmented with *Rootine*, and roots marked in red are only segmented with *RootForce*.

The small roots are similarly detected as for the cassava plants. Specifically for the storage roots, the segmentation with *Rootine* has some issues. This leads to the fact that the roots are not connected, and it was impossible to get rid of the surrounding soil as presented in Figure 8. The main challenge of *Rootine* is the segmentation of the storage roots. Even when using a large value for the root diameter, it was not possible to segment the storage roots.

#### 4.2.2. Comparison between *RootForce* and Manual Segmentation for Time Series

**Figure 9:**
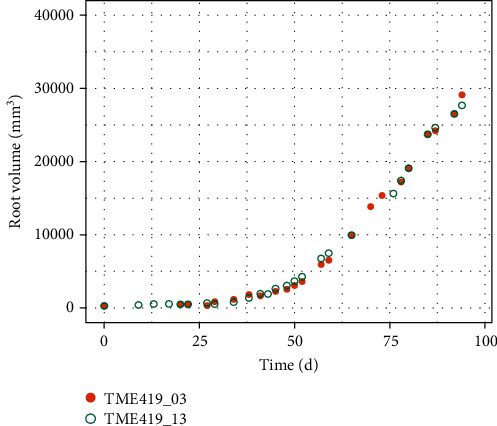
Comparison between semiautomatic (orange dots, TME419_03) and manual (green dots, TME419_13) segmentations for cassava TME419 plant #03. The computed root volume (in %) is presented over 100 days after planting for both segmentation approaches.

The 3D images obtained by *RootForce* (Figure 7(c)) and manual segmentation (Figure 7(d)) for plant #03 show minor differences for day 20 only. For a more detailed comparison, the extracted total root volumes *V*_root_ are plotted for the growth time of 100 days in Figure 9 for the manual (green) and semiautomatic (orange) segmentation approaches. The segmentations are conducted for the first 100 days after planting. Being directly visible, the number of roots missing in the semiautomatic approach (e.g., around day 60) are almost diminishing and almost unnoticeable. One main reason for the similarity of the results of both segmentation approaches is the focus on the storage roots by using binned reconstructed volumes, which represent the greatest volume fraction and can be clearly segmented. Lateral roots were neglected in this issue.

#### 4.2.3. Growth Tracking of Cassava Storage Root Development

**Figure 10:**
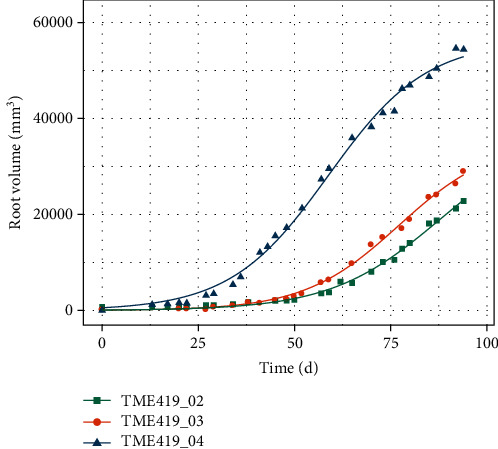
Root volume development over time for cassava TME419 over 100 days after planting for all three plants in comparison: green rectangle, plant 02; orange circle, plant 03; blue triangle, plant 04.

Figure 10 depicts the root volume development over 100 days in comparison. As can be seen, the storage root bulking starts 27 days for plant #02 (denoted in green squares), 34 days for plant #03 (orange dots), and 17 days for plant #04 (blue triangles) after planting. Plant #01 (not depicted) starts storage root bulking at day 92, delayed due to a kinked main stem and is not analyzed here. The root volume development for plants #02 and #03 shows some similarities in behavior and bulking (after about 30 days after planting) with growth rates of 474 mm^3^/d (plant #02) and 541 mm^3^/d (#plant 03). For plant #04, an accelerated subroot bulking was indicated already 17 days after planting. Accordingly, the root volume development and growth rate (816 mm^3^/d) is increased.

In summary, the storage root analysis for cassava plants delivers solid results on storage root development and enables the extraction of individual growth rates during storage root bulking. The root analysis results allow for a comparison of different plants, genotypes, and conditions, which directly affect root growth [20, 29]. The same parameter set (see Section 3.3) was used for the semiautomatic root segmentation of all cassava plants, so it can be stated that the extraction works for various root diameters.

During the cassava research study, over 500 cassava scans were analyzed, underlining the importance of automated image analysis already discussed by Atkinson et al. in 2019 [1], Zappala et al. in 2013 [29], and van Dusschoten et al. in 2016 [20] who also applied 3D root imaging for monitoring root system growth over time.

## 5. Discussion

In summary, for the delineation of both types of plants (beans and cassava), it can be stated that the semiautomated segmentation finds especially more small roots and they are better connected to the main root system. This fact becomes also statistically measurable, when the calculated root length is compared to WinRHIZO data (obtained from the previous work [4]). To this end, the parameters of *RootForce* can be adapted to the interesting structures, them being either huge storage roots or thin lateral roots. For a more detailed view, the segmentation of lateral roots in the range of the resolution is possible but linked to longer computing time and, in some circumstances, postprocessing. The importance of lateral root visualization in dependency from the research aspect is also underlined by the publication of Bao et al. in 2014 [30], who visualized the lateral roots of maize seedlings on a comparable resolution of 22 *μ*m to show the influence of surrounding conditions on root architecture development.

The fast segmentation of approximately 7-15 minutes per plant scan (depending on the downscaled binning mode) is sufficient for various types of investigation of the root architecture. This is especially true if 3D + *t* CTX data is addressed and most important in the case for nondestructive longitudinal growth experiments for plant phenotyping, where the huge amount of 3D + *t* image data cannot be assessed manually. Furthermore, if the segmentation parameters have been adjusted once to the type of image data, plant, and soil, they can be held constant over all successive plants and all time points. The 3D + *t* data segmentation can then be automatically assessed over a database approach. This necessity of batch-mode analysis is also supported by Atkinson et al. [1], who state that “As high-throughput image capture of root systems has become mainstream and generates ever larger datasets, there is a requirement for fast and accurate software solutions to reliably derive traits”.

## 6. Conclusions

Global root features automatically extracted from high-resolution CTX scans are a valuable tool compared to destructive sampling, which leads to the loss of finer lateral root architectural features and usually measures only a snapshot of root development [7, 31, 32]. For example, in 2015, Foereid [33] investigated the root quantification from X-ray data and names the lateral roots as one important main factor for missing correlations. The quantification becomes less reliable for these smaller roots; therefore, the scanning resolution should be always adapted to the research aim and root system.

Thus, in order to support the image assessment of large scale CTX data of various plants, a variable and flexible automated root segmentation approach (*RootForce*) was presented and evaluated on bean and cassava plants under different aspects.

Therefore, bean plants were recorded at high resolution to focus on lateral roots. The result of semiautomatic segmentation is comparable to manual image processing, whereby semiautomatic processing is more precise due to better connection and definition of the roots. The fact that full-resolution segmentation has been made available through the possible processing of large datasets allows the segmentation of lateral roots down to 0.03 mm diameter.

Cassava plants were planted in large pots (with a diameter of ∅ = 200 mm) and were scanned over a period of 100 days in order to investigate their storage root development and calculate the growth rate during storage root bulking. Segmentation of these time series (yielding 3D + *t* data) was done completely in batch mode, as the parameters of the delineation process could be held constant over all data and time points, despite changes in soil moisture and compactness over time. Thus, differences between plants, genotypes, and growth conditions can be studied. The proposed semiautomated segmentation shows only negligible differences for the cassava plants in comparison to manual image processing.

## Figures and Tables

**Table 1:** Parameters for the CTX imaging for the bean and cassava plants.

	Bean plants	Cassava plants
Tube	FXE 225.99 X-ray tube	Comet 225 HP/11 X-ray tube
Detector	PerkinElmer XRD 1620 flat panel detector	Fraunhofer XEye 2020 flat panel detector
Spatial resolution of detector (pixel^2^)	2048 × 2048	2040 × 2048
Frame rate (images/second)	2	2.85
Bit-depth (bits)	14	16
Acceleration voltage (kV)	220	175
Current (*μ*A)	180	4,000
Exposure time (ms)	499	350
Filter	N/A	1 mm copper
Number of projections	1,200	1,600
Effective dose	Not measured	200 mGy
Cubic voxel size (*μ*m)	28.15, 34 (depending on pot size)	87.7
Mean scan time per plant	2-3 times: 10 minutes	9 minutes

**Table 2:** Global features calculated from *RootForce* for the segmented roots are shown in Figures 3(b) and 4(b).

	Small pot ∅ = 34 mm	Medium pot ∅ = 56 mm
Total root volume *V*_root_ (mm^3^)	1290.4	1333.3
Total root length *L*_root_ (mm)	4597.6	4010.7
25% Rootmass quantile depth (mm)	10.5	36.5
50% Rootmass quantile depth (mm)	20.3	62
75% Rootmass quantile depth (mm)	34.3	77.7
90% Rootmass quantile depth (mm)	44.6	85.1
Form fraction *F* (in %)	6.4	2.4
Minimum root angle *θ*_min_ (in °)	83.1	87.2
Maximum root angle *θ*_max_ (in °)	89.6	89.4
Mean root angle *θ*_mean_ (in °)	88.7	88.6
Mean root density (gray intensity values)	2767.7 ± 405.3	3179.7 ± 422.8
Mean soil density (gray intensity values)	3168.4 ± 1381.1	3660.7 ± 1535.5
Earth/roots density relation factor (in %)	114.5	115.1

**Table 3:** Parameter sets *P* used by *RootForce* for analysis of the root CTX root da data.

Values	Bean plant small pot	Bean plant medium pot	Cassava plants
Attenuation value range	(0.11,…, 0.24)	(0.11,…, 0.24)	(0.08,…, 0.2)
*D*_root_ (voxel)	2, 2.5, 3, 3.5, 4, 5, 6	2, 2.5, 3, 3.5, 4, 5, 6	1, 1.5, 2, 3
*θ*_1_ (gray values)	400	600	60
*θ*_2_ (gray values)	2,500	2,000	65,000
*V*_min_ (mm^3^)	1	1.6	5

**Table 4:** Root traits calculated from CT images of the segmented volumes are shown in Figures 3(a) and 3(b) and Figures 4(a) and 4(b).

Segmentation	Small pot (∅ = 34 mm)		Medium pot (∅ = 56 mm)	
	Manual	Semiautomatic	Manual	Semiautomatic
Resolution (*μ*m)	56.3	56.3	68	68
Total root volume *V*_root_ (mm^3^)	1568	1290	1228	1333
Total root length *L*_root_ (mm)	2775	4598	2787	4011
Percent of WinRHIZO (%)	69	114	57	83

## Data Availability

An evaluation version of the *RootForce* package as well as some reference images can be obtained from the senior author (SG).
